# Notes from the Field: *Clostridium perfringens* Outbreak at a Catered Lunch — Connecticut, September 2016

**DOI:** 10.15585/mmwr.mm6635a3

**Published:** 2017-09-08

**Authors:** Vivian H. Leung, Quyen Phan, Cynthia E. Costa, Christina Nishimura, Kelly Pung, Liz Horn, Lynn Sosa

**Affiliations:** ^1^Epidemic Intelligence Service, CDC; ^2^Connecticut Department of Public Health; ^3^Minnesota Department of Health.

In September 2016, the Connecticut Department of Public Health was notified of a cluster of gastrointestinal illnesses among persons who shared a catered lunch. The Connecticut Department of Public Health worked with the local health department to investigate the outbreak and recommend control measures. Information about symptoms and foods eaten was gathered using an online survey. A case was defined as the onset of abdominal pain or diarrhea in a lunch attendee <24 hours after the lunch. Risk ratios (RRs), 95% confidence intervals (CIs), and Fisher’s exact p-values were calculated for all food and beverages consumed. Associations of food exposures with illness were considered statistically significant at p<0.05.

Among approximately 50 attendees, 30 (60%) completed the survey; 19 (63%) respondents met the case definition. The majority of commonly reported symptoms included diarrhea (17 of 18), abdominal pain (15 of 16), and headache (7 of 15). The median interval from lunch to illness onset was 5.3 hours (range = 0.4–15.5 hours) for any symptom and 7 hours (range = 2.5–13 hours) for diarrhea. Analysis of food exposures reported by 16 ill and 10 well respondents (four respondents did not provide food exposure information) found illness to be associated with the beef dish (RR = undefined; CI = 1.06–∞; p = 0.046) (Table). All 16 ill respondents reported eating the beef. Coffee was also associated with illness; however, all 13 coffee drinkers who became ill also ate the beef. Eating cake approached significance (p = 0.051); all 10 cake eaters who became ill also ate the beef.

**TABLE:** Associations between illness and food exposures reported by attendees at a catered lunch — Connecticut, September 2016

Food/Drink exposure	Ill persons (n = 16)		Well persons (n = 10)		Risk ratio (95% CI)	P-value
	No. who ate item	No. who did not eat item	No. who ate item	No. who did not eat item		
Tripe	12	4	5	5	1.59 (0.72–3.51)	0.234
Fish	9	7	3	7	1.50 (0.81–2.78)	0.248
Pork	10	6	5	5	1.22 (0.64–2.34)	0.689
Chicken	9	7	6	4	0.94 (0.51–1.73)	1.000
Beef	16	0	7	3	—* (1.06–∞)	0.046^†^
Noodles	11	5	7	3	0.98 (0.51–1.88)	1.000
Mixed vegetables	8	8	4	6	1.17 (0.64–2.14)	0.702
Spring rolls	14	2	7	3	1.67 (0.55–5.08)	0.340
Cake	10	6	2	8	1.94 (1.01–3.75)	0.051
Pudding	7	9	3	7	1.24 (0.69–2.25)	0.683
Yam dessert	10	6	4	6	1.43 (0.74–2.75)	0.422
Rice	15	1	9	1	1.25 (0.30–5.17)	1.000
Grapes	9	7	5	5	1.10 (0.59–2.04)	1.000
Mango salad	6	10	4	6	0.96 (0.51–1.81)	1.000
Muffin	5	11	1	9	1.52 (0.89–2.58)	0.352
Bagel	8	8	2	8	1.60 (0.90–2.86)	0.218
Coffee	11	5	2	8	2.20 (1.06–4.55)	0.041^†^
Juice	5	11	2	8	1.23 (0.67–2.26)	0.668
Water	15	1	10	0	0.60 (0.44–0.83)	1.000
Soda	4	12	2	8	1.11 (0.57–2.17)	1.000

**Abbreviations:** CI = confidence interval; RR = risk ratio.

* The risk ratio is undefined because the calculation involves dividing by zero.

^†^ Statistically significant finding.

The caterer had begun preparing all dishes the day before the lunch. Meats were partially cooked and then marinated in the refrigerator overnight. In the morning, they were sautéed 2 hours before lunch. Inspection of the facility found the limited refrigerator space to be full of stacked containers that were completely filled with cooked food, disposable gloves that appeared to have been washed for reuse, and a porous wooden chopping block.

The caterer’s four food workers reported no recent illness. Stool specimens from the food workers and from four ill attendees all tested negative for norovirus, *Campylobacter*, *Escherichia coli* O157, *Salmonella*, and *Shigella* at the Connecticut State Public Health Laboratory. All eight specimens were sent to the Minnesota Department of Health Public Health Laboratory, where additional testing was available. Two specimens from food workers were positive for enterotoxigenic *Escherichia coli* by polymerase chain reaction, but no enterotoxigenic *E. coli* colonies were isolated. Seven specimens (four from food workers and three from attendees) were culture-positive for *Clostridium perfringens*, and specimens from all attendees contained *C. perfringens* enterotoxin. Pulsed-field gel electrophoresis of 29 *C. perfringens* isolates from the culture-positive specimens found no matches among attendee isolates, but demonstrated a single matching pattern between two food worker specimens. No leftover food items were available for testing.

*C. perfringens*, a gram-positive, rod-shaped bacterium, forms spores allowing survival at normal cooking temperatures and germination during slow cooling or storage at ambient temperature (*1*). Diarrhea and other gastrointestinal symptoms are caused by *C. perfringens* enterotoxin production in the intestines. Vomiting is rare and illness is usually self-limited, although type C strains can cause necrotizing enteritis (*1*). 

Symptoms reported were consistent with *C. perfringens* infection, with a predominance of diarrhea, and median diarrhea onset time was at the lower end of the typical *C. perfringens* incubation period (6–24 hours) (*1*). *C. perfringens* enterotoxin detection in the stool of two or more ill persons confirms *C. perfringens* as the outbreak etiology (*2*). Both *C. perfringens* and enterotoxigenic *E. coli* can colonize asymptomatic persons (*3*,*4*), which might explain the presence of these pathogens in the stools of asymptomatic food workers. Pulsed-field gel electrophoresis did not identify the *C. perfringens* strain responsible for the outbreak, but findings add to the evidence for a wide variety of *C. perfringens* strains, not all producing *C. perfringens* enterotoxin (*5*).

*C. perfringens* outbreaks are typically associated with improper cooling or inadequate reheating of contaminated meats (*1*), which might have occurred with the beef dish. The restaurant was advised about the need for adequate refrigeration and best practices for cooling foods, including using stainless steel rather than plastic containers, avoiding filling containers to depths exceeding two inches, avoiding stacking containers, and ventilating hot food. Upon follow-up inspection, staff members discarded disposable gloves after one use, used only food-grade cutting boards, and maintained proper food temperatures for hot holding, cold holding, cooling, and reheating, as outlined in the Food and Drug Administration Food Code.

An estimated 1 million illnesses in the United States each year are attributable to *C. perfringens*, but fewer than 1,200 illnesses are reported annually with *C. perfringens* outbreaks (*6*). *C. perfringens* testing is not routine for foodborne outbreaks; even if testing is unavailable, *C. perfringens* should be considered when improper cooling, inadequate reheating, and improper temperature maintenance of meat are identified.
