# *Erratum*: Vol. 66, No. 35

**DOI:** 10.15585/mmwr.mm6637a10

**Published:** 2017-09-22

In the report “Notes from the Field:* Clostridium perfringens* Outbreak at a Catered Lunch — Connecticut, September 2016,” on page 940, the sixth sentence of the second paragraph should have read “Coffee was also associated with illness; however, **all 13 coffee drinkers also ate the beef**.”

